# Listerial Rhombencephalitis in an Immunocompetent Woman

**DOI:** 10.1155/2014/674321

**Published:** 2014-07-13

**Authors:** Piotr Czupryna, Agata Zajkowska, Adam Garkowski, Sławomir Pancewicz, Katarzyna Guziejko, Anna Moniuszko, Joanna Zajkowska

**Affiliations:** Department of Infectious Diseases and Neuroinfections, Medical University of Białystok, Żurawia 14, 15-540 Białystok, Poland

## Abstract

Listeriosis usually affects immunocompromised patients including elderly people and pregnant women, but it may also affect otherwise healthy individuals. In our report, we present a case of a rare and very severe form of listeriosis-rhombencephalitis in a 61-year-old female with no history of immunosuppression, who, because of history, clinical picture, and laboratory results as well as negative cultures, was at first diagnosed with viral encephalitis. This paper underlines that *Listeria monocytogenes* infection should be taken into consideration in case of lymphocytic encephalitis even in immunocompetent patients. Typical MRI picture may be crucial in establishing a proper diagnosis as the lab results may be misleading.

## 1. Introduction

*Listeria monocytogenes*, the causative agent of human listeriosis, is a gram-positive facultatively intracellular bacterium.* Listeria* grows at 37°C but can also grow slowly at 4°C.* Listeria monocytogenes* is widespread in the environment and has been isolated from oil, water, and vegetables as well as many animals including birds, crustaceans, and fish. The main route of infection is ingestion of contaminated food; however, the infection can be also transmitted from animals to humans and from humans to humans [1–3]. There is a possibility of fetus infection via the placenta [1, 4].

The disease occurs sporadically although in recent years an increase in incidence in many European countries is noted, mostly in elderly people [1]. The incidence of listeriosis is 0.29 cases per 100,000 persons in USA [5]. In Europe, it varies from 0 to 0.75 cases per 100,000 persons and is higher in countries with statutory reporting of cases and surveillance through a national reference laboratory [6, 7]. In Poland, in 2012, the incidence was 0.13 per 100,000 persons [8].

Listeriosis usually affects immunocompromised patients including elderly people and pregnant women, but it may also affect otherwise healthy individuals. Two forms of the disease can be differentiated: noninvasive gastrointestinal listeriosis and invasive listeriosis. Immunocompetent people usually develop noninvasive form of listeriosis which presents as febrile gastroenteritis [1, 9]. Invasive listeriosis affects immunocompromised adults and may manifest as septicaemia or meningoencephalitis [1].

One of the main risk factors for infection is lack of cell-mediated immunity as the bacterium is an intracellular pathogen.* Listeria* is able to penetrate phagocytic and nonphagocytic cells, thus avoiding the host immune system. Bacteria enter the epithelial cells of the intestine and then enter the bloodstream.

The overall mortality of listeriosis is 20–30% [9] and in listerial brainstem encephalitis may be as high as 51% [10].

In our report, we present a case of a rare and very severe form of listeriosis-rhombencephalitis in a 61-year-old female with no history of immunosuppression.

## 2. Case Report

A 61-year-old female with a 2-week history of headache, vertigo, nausea, and drowsiness was admitted to the ward with a suspicion of meningitis. The patient was living in an endemic area of tick-borne diseases (tick-borne encephalitis and Lyme disease). Also, patient's family stated that she might have been bitten by ticks recently.

Fever up to 39°C appeared 1 day before hospitalization. During this 2-week period of time, she was twice consulted in the neurological ER. Two CT examinations of the brain did not show any focal lesions or hemorrhage.

On admission, the patient was conscious (12 points in GCS). In the physical examination, dehydration, fever (38.8°C), and neck stiffness were stated. Laboratory tests showed only moderate hyponatremia. CRP and WBC were in normal range. Lumbar puncture was performed and the CSF had inflammatory features (pleocytosis 49 cells/ul, with lymphocyte predominance and protein concentration 67.7 mg/dL).

The history of tick bites, biphasic course of the disease, and CSF examination suggested TBE or neuroborreliosis. Therefore, the patient in the initial treatment received 20% mannitol and ceftriaxone. However, serological tests excluded tick-borne encephalitis and Lyme disease. On the 3rd day of hospitalization, consciousness disturbances appeared (GCS 10). Herpes etiology was considered; acyclovir and dexamethasone were administered.

With an exception of the fourth day of hospitalization, the patient was afebrile.

On the fifth day of hospitalization, the patient was unconscious, with no response to stimuli, eyes deviated to the right, and narrow pupils with weak response to light (GCS 8). To exclude brain stroke, MRI was performed.

**Figure 1:**
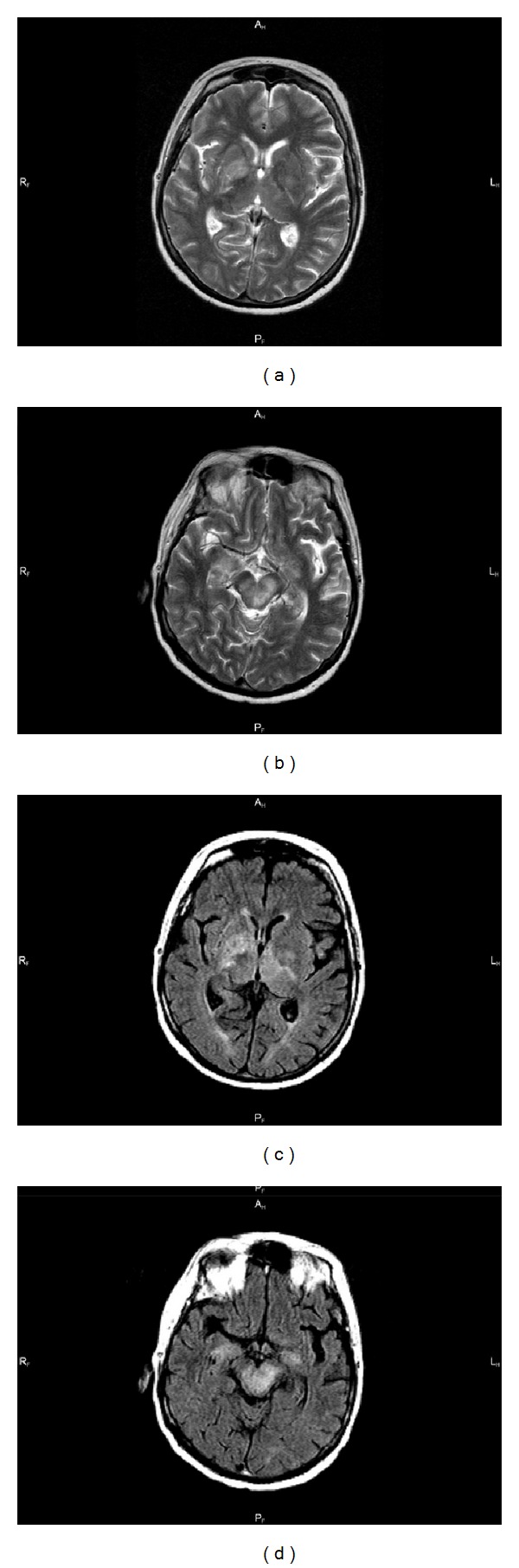
Brain MRI in T2 (a, b) and FLAIR (c, d).

In MRI of the brain, in T2 images, nonspecific inflammatory lesions were observed (irregular hyperintensive lesions in pallidum, internal capsules, cerebral pedunculi, and right cerebellum hemisphere) (Figure 1). Because of increasing dyspnoea (SpO2, 73%) and general severe condition of the patient, she was transferred to ICU.

Six days later, in blood culture, ampicillin-resistant* Listeria monocytogenes* was identified (first blood cultures were negative). The bacteria were susceptible to meropenem.

At ICU, for the patient, meropenem was administered. Patient required mechanical ventilation; blood pressure was stabilized with catecholamines.

Despite intensive treatment, she did not regain consciousness and remained in a vegetative state. On the 23rd day of hospitalization, tracheostomy was performed and, on the 25th day, gastrostomy was performed. The patient was transferred to the hospice for further care.

## 3. Discussion

Central nervous system (CNS) listeriosis accounts for 7.4 to 16 cases in 1 million individuals (47% of* L. monocytogenes* infections) [11].

There are at least 3 different mechanisms that enable* Listeria monocytogenes* invasion of CNS: transport across the blood-brain or blood-choroid barriers within leukocytes, direct invasion of endothelial cells by extracellular blood-borne bacteria, and retrograde (centripetal) migration into the brain within the axons of cranial nerves [12, 13]. In the CNS,* Listeria* penetrates microglia, astrocytes, and oligodendrocytes. Encephalitis is established by direct cell-to-cell spread. The infection leads to necrosis and focal hemorrhages in the brain with meningoencephalitis, microabscesses, vasculitis, and perivascular lymphocytic infiltration [3].

Listerial rhombencephalitis (brain stem encephalitis) is a rare form of listeriosis (9% of CNS listeriosis cases) that usually affects immunocompetent people [11]. In various studies, patients with no history of immunosuppression account for 42–92% of the examined patients with listerial rhombencephalitis [14–16]. The disease has usually biphasic course [10]. The prodromal phase lasts for 0 to 16 days and manifests with fever, headache, vomits, or nausea. It is followed by neurological phase. This is similar to viral meningitis (e.g., TBE).

The most common neurological symptoms are cranial nerves pareses (most commonly VI and VII), cerebellar symptoms, motor and sensory deficits, and consciousness disturbances. In 41% of cases, the disease may lead to respiratory failure [14, 17, 18].

CSF examination usually reveals moderate pleocytosis (median 110 cells/mm^3^) with lymphocyte predominance and normal glucose concentration. White blood cells count and CRP may remain low [14, 15, 17]. Therefore, the disease may be easily mistaken for viral meningitis especially if CSF cultures are negative—as it was in the described case.

Blood cultures are positive in 61% of cases and CSF cultures are positive in 11–41% [14]. Therefore, cultures should be taken frequently, even after implementation of antibiotic treatment. Serology is not useful for diagnosis of acute cases, although listeriolysin O antibodies have been used to identify patients with noninvasive illness. As far as diagnostic imaging is concerned, MR imaging is crucial for detection of parenchymal lesions and early diagnosis of this illness: patchy signal hyperintensity throughout the medulla and cerebellar peduncles on T2 weighted images, always in association with a hypointense dot, and numerous gadolinium-enhanced microabscesses in the rhombencephalon [18, 19].

MRI is superior to CT in listerial rhombencephalitis diagnosis [14, 20, 21]. In case of our patient, age (61 years) was the sole risk factor as the patient did not suffer from any chronic diseases. The initial CSF examination and lab tests, negative cultures from CSF, and serum as well as history of tick bite were strongly suggestive of tick-borne encephalitis, which is one of the most frequent causes of meningitis and encephalomeningitis in our region. Because of the deteriorating clinical state of the patient and appearance of consciousness disturbances, another etiology was favored and acyclovir and antibiotics were administered.

In the course of the disease, the patient had CT of the brain twice and no abnormalities were found. When patient state exacerbated and MRI was performed, the picture was typical for listerial rhombencephalitis.

The differential diagnosis for rhombencephalitis should include MS, Behcet disease, paraneoplastic syndrome, and other infections (EBV and* Mycoplasma*) [22, 23].

The drugs of choice in listerial infections are ampicillin and penicillin [14]. However, the strain of* Listeria monocytogenes* cultured from patient's blood was ampicillin resistant. In a study of Morvan et al. which assessed antibiotics resistance changes in human isolates in years 1926–2007, a significant increase in median MICs of aminopenicillins was observed [23].

A study of Srinivasan et al. showed that ampicillin resistant strains of* Listeria* are frequent in the environment. 92% of* Listeria* strains isolated from dairy farms were ampicillin resistant [24].

## 4. Conclusions

*Listeria monocytogenes* may affect immunocompetent patients and listerial encephalitis may, at first, resemble viral encephalitis. Therefore, listeriosis should be taken into consideration in case of lymphocytic encephalitis.

Typical MRI picture may be crucial in establishing a proper diagnosis as the lab results may be misleading.

Blood cultures should be taken frequently even after antibiotic treatment.

The drugs of choice for listerial infections are ampicillin and penicillin; however, some strains may be resistant.
